# Workplace management practices and quiet quitting among primary healthcare workers: a sequential-parallel mediation analysis

**DOI:** 10.3389/fpubh.2026.1903586

**Published:** 2026-08-06

**Authors:** Lanxin Xia, Zhikai Yu, Si Fan, Qianqian Xu

**Affiliations:** 1 School of Accounting, Wuhan College, Wuhan, Hubei, China; 2 School of Chinese Language and Literature, Hubei University, Wuhan, Hubei, China; 3 School of Medicine and Health Management, Tongji Medical College, Huazhong University of Science and Technology, Wuhan, Hubei, China

**Keywords:** Hawthorne experiments, management practice, managerial factors, primary healthcare workers, quiet quitting

## Abstract

**Background:**

The quiet quitting phenomenon, a new emerging and hidden turnover behavior, is prevalent among primary healthcare (PHC) workers. Addressing their quiet quitting requires exploring possible managerial factors of management practice such as goal-setting, organizational centralization, organizational culture and incentives in the workplace, which are rarely jointly considered but offering great value for the construction of healing workplaces amid current medical reform. This study explores the associations between management practices in primary healthcare institutions and quiet quitting among PHC workers. We further construct a sequential-parallel mediation model to test two distinct indirect pathways triggered by goal-setting: the first is a sequential chain mediation pathway via organizational centralization and organizational incentives, and the second is a single mediation pathway via organizational culture.

**Methods:**

This is a two-wave cross-sectional study surveyed PHC workers on-site from all 11 primary healthcare institutions in a county where the management abilities have been strengthened in the context of health poverty revitalization. Measurements of managerial factors and quiet quitting are based on their classic scale with acceptable reliability and validity. Structural equation modeling (SEM) and mediation analysis were used for statistical analysis.

**Results:**

The mechanism shows that goal-setting have significant negative association with quiet quitting through two pathways. Firstly, goal-setting has a significantly positively association with organizational centralization (β = 0.774, *P* < 0.05). Organizational centralization has a positive association with incentives (β = 0.742, *P* < 0.05) and incentives are negatively associated with quiet quitting (β = −0.229, *P* < 0.05). Secondly, goal-setting has a positive association with organizational culture (β = 0.787, *P* < 0.05), organizational culture is negatively associated with (β = −0.649, *P* < 0.05) quiet quitting. Final mediation effect analysis shows that the association between goal-setting and quiet quitting through organizational culture (indirect effect = −0.511, *P* < 0.01) is much greater than combined association between organizational centralization and incentive (indirect effect = −0.132, *P* < 0.01).

**Conclusions:**

This study offers key insights into quiet quitting among primary healthcare workers, highlighting that reasonable managerial design may help reduce quiet quitting and protect employees' well-being.

## 1 Introduction

The quiet quitting among primary healthcare (PHC) workers now is attracting more attention. PHC workers such as general practitioners play an important role in safeguarding health rights of the citizens as well as controlling the medical expenditure (1). However, workforce shortages and burnout have become persistent challenges in primary healthcare systems for decades (2, 3). Many PHC workers increasingly seek less demanding positions to reduce work pressure. Accompanying with these high turnover rate and burnout, an emerging and hidden phenomenon of quiet quitting among PHC workers has just attracted great attention from the academia (4).

Quiet quitting refers to the phenomenon of workers limiting their extra work efforts to basic job requirements which by nature is a kind of psychological disengagement from work with concealment (5). According to a recent Gallup survey, half of the American work-force are quiet quitting their work, which leads to a significantly reduction in employee productivity than in many industries (6). The quiet quitting among PHC workers also attacks medical system. Some studies have shown that a large percentage of PHC workers are quiet quitters (7, 8). A cross-sectional study shows that nearly 57.9% of PHC workers in Greece were quiet quitters (9), which leads to a decline in healthcare quality and productivity. Recognizing that managerial practices are key to primary healthcare for both preventing quiet quitting and fostering supportive work environments, many global health policies now integrate management strategies in organization, incentives, and goal-setting to tackle this issue.

From the perspective of salary incentives, PHC workers in United Kingdom are allowed to work in Multi-Clinic Practice to increase their income and then achieve the aim of improve supply of medical service (10). China has also implemented a number of measures under perspective of organizational incentives such as title promotions, subsidization and so on to improve PHC workers' service capacity and working enthusiasm (11). Overall, managerial factors shaping the quality of work environments are associated with employees' quiet quitting behavior. Given the high prevalence of quiet quitting and its great academic importance, exploring multiple managerial influencing factors is essential for designing healthier, healing-oriented workplaces.

Numerous studies have established robust associations between organizational management practices and the work-related outcomes of primary healthcare workers (12, 13). Existing research commonly examines managerial factors such as target setting, operational management, and performance monitoring (13–15). To evaluate the overall management effect, scholars usually combine these dimensions into a unified composite score. Nevertheless, this aggregated index approach masks functional disparities across different managerial dimensions. Such aggregation masks how managerial structures interact and cofunction within primary healthcare institutions. In particular, the internal relationships among managerial mechanisms and their joint influence on employee behavioral disengagement remain insufficiently understood.

This study makes three distinct contributions to the literature. First, this study enriches the existing literature by situating the analysis within China's primary healthcare context. Most prior research has drawn on evidence from Western organizations or large hospitals, leaving a notable gap in understanding quiet quitting and managerial mechanisms among frontline healthcare workers operating under China's hierarchical public health system. Second, this study departs from the conventional composite scoring approach to management practices. Instead, it identifies four interrelated managerial elements embedded in management practice, allowing for a more fine-grained examination of their mutual associations. These associations are often obscured when scholars rely solely on aggregated overall scores. Third, in terms of analytical strategy, this study constructs a sequential parallel mediation model to systematically disentangle interrelationships across managerial dimensions and clarify how these factors are jointly associated with quiet quitting among primary healthcare workers through specific pathways.

## 2 Theoretical background and hypotheses development

### 2.1 Theoretical framework

There are numerous qualitative research about the influence of managerial factors on working behavior such as goal-setting of planning, organization, incentives (16, 17). From classic management theory, the planning is the first managerial functions among five basic management functions and goal-setting is a key component of planning, that refers to clear and attainable goal guidance given to employees by the organization (18, 19). Organizational centralization is the concentration of decision-making power in a particular leader or manager at the top of the organizational hierarchy (16).

To understand how managerial factors are associated with quiet quitting among PHC workers, the Job Demands-Resources (JD-R) theory provides a core theoretical framework for explaining the driving mechanism of employees' negative work behaviors in the workplace (20). This JD-R model classifies workplace characteristics into two core dimensions: job demands and job resources. Job demands reflect ongoing physical and psychological workload pressures within primary healthcare settings, which incur chronic psychological and emotional costs and are further associated with negative work attitudes among PHC workers. In contrast, work resources encompass the assets available to organizations and management, such as appropriate goal-setting and sound governance arrangements; these resources help to accomplish tasks, alleviate work-related stress, mitigate the adverse effects of high work demands, and promote employees' professional development.

In the context of Chinese primary healthcare, quiet quitting can be conceptualized as a disengagement response to perceived resource inadequacy. Managerial factors such as clear goal-setting, appropriate organizational centralization, effective incentives, and supportive organizational culture function as job resources that buffer the negative effects of demands and sustain employee engagement (21).

Zhao et al. (22) drawing on the JD-R model, demonstrate that perceived organizational support, which can be considered as one of the work resources, positively predicts the confidence of primary care general practitioners in China in managing multimorbidity. Furthermore, a cross-sectional study of primary care workers in Guangzhou confirms that work resources, such as particularly social support, job autonomy, career development opportunities and so on, positively predict organizational citizenship behavior (20). Within this theoretical framework, “quiet quitting” can be understood as the result of a perceived imbalance between the job demands placed on primary healthcare workers and the management resources available to them.

### 2.2 Conceptual framework and core managerial constructs

The foundation of this study's management assessment framework is the well-validated World Management Survey (WMS) (23), renowned for its rigorous measurement of practices across diverse sectors. However, as the original WMS dimensions (Operations Management, Performance Monitoring, Target Setting, Leadership Management, and Talent Management) were largely derived from empirical work in manufacturing and education, a contextual adaptation was necessary to enhance their relevance to primary care settings. These settings are typically characterized by decentralized structures, significant professional autonomy, and multifaceted objectives.

Our adaptation was informed by both theoretical and methodological considerations. Theoretically, we employed the management process theory as a conceptual lens. This perspective, which deconstructs management into a set of interrelated core functions such as planning, organizing, leading, and controlling, provided a logical basis for refocusing and reinterpreting the WMS items. Methodologically, we followed the precedent set by McConnell et al. (24), who successfully translated and validated the WMS methodology, originally rooted in economics, for application in healthcare environments.

The original WMS framework covers five core areas of management: Operations Management, Performance Monitoring, Target Setting, Leadership Management, and Talent Management (12). Notably, the WMS framework does not have a fixed dimensional structure across different research contexts; prior Chinese studies have adopted varying dimensional configurations depending on the specific context, with some retaining the original four dimensions and others extracting five factors (14, 25). Drawing on this flexibility and the characteristics of primary healthcare settings, we reconfigured the original dimensions into four managerial factors: goal-setting, organizational centralization, organizational culture, and incentives.

Guided by our theoretical and methodological framework, we reconfigured these original dimensions into four managerial factors relevant to primary care, as detailed in Supplementary Table S1. The original 1–5 scoring scale was retained. To clarify the conceptual linkage between our adapted framework and the original WMS, we provide a conceptual mapping in Supplementary Table S6. This table illustrates how each of our four managerial factors draws on specific WMS domains while also incorporating theoretical insights from established management theories relevant to primary healthcare. It should be noted that our measures are not direct translations of WMS items. Instead, we adapted the conceptual scope of the WMS domains and operationalized them using items from validated scales in the literature. To ensure transparency in our adaptation process, Supplementary Table S1 presents a detailed mapping of how the original WMS dimensions and items were transformed into these four final constructs, along with the rationale for each adaptation.

### 2.3 Hypotheses development

In the field of primary healthcare (PHC) in China, particularly in the context of top-down initiatives to promote health-related poverty alleviation and rural revitalization, goal-setting plays a role through a governance logic characterized by strengthened organizational centralization. Specifically, since 2015, China has established a nationwide, systematic and coordinated governance framework for health-related poverty alleviation, which primarily encompasses three key policy directions: the precise identification of people living in poverty, the enhancement of healthcare coverage, and the optimization of healthcare resource allocation (26). Empirical data from rural areas in China show that the effectiveness of health-related poverty alleviation interventions depends on strict top-down oversight and a high degree of expert control. This is primarily because local primary healthcare organizations lack the capacity to implement reforms proactively and independently (27). At the same time, the targeted poverty alleviation initiative did not rely on decentralized, locally driven measures, but was instead implemented through a series of centrally coordinated economic, social and healthcare measures (26). As another health initiative coordinated and promoted by the central government to support the “Healthy China 200” strategy, primary healthcare system reforms have been shown to be critically dependent on workforce stability, as studies indicate that factors such as work intensity, salary satisfaction, and burnout among primary public health workers are significantly correlated with their turnover intention (28). Under the logic of the aforementioned system, the setting of objectives has further enhanced the degree of organizational centralization by strengthening hierarchical control; the core features of this logic are reflected in a vertical governance model, legally binding policy directives, and a centrally coordinated implementation mechanism. Previous research revealed goal-setting could promote organizational development such as strengthen organizational centralization and construct positive organizational culture (29, 30). Another study also found that goal-setting can strengthen organizational centralization by enhancing employee's work incentives (31). Thus, goal-setting provides a theoretical rationale for expecting its positive association with organizational centralization. Therefore, this study proposes the first hypothesis:

Hypothesis 1: Goal-setting has a positive association with organizational centralization in primary healthcare institutions.

Incentives as a powerful management tool can enable employees to behave expected by organization and usually are linked with organizational centralization. Ghana's decentralized health system presents a representative illustration. District health managers are endowed with greater decision-making autonomy, which serves as an effective incentive to enhance health workers' motivation and job satisfaction and reduce their turnover intention (32). This evidence reveals that properly designed organizational structures, be they centralized or decentralized, can deliver robust incentives to optimize workforce performance in primary healthcare. Another case is the positive incentive role leaded by centralized management in coordinating China's economic development and social stability, especially in health poverty alleviation (33). However, relative research about the association between organizational centralization and incentives are rare in context of primary healthcare. So, this study proposes the second hypothesis:

Hypothesis 2: Organizational centralization has a positive association with organizational incentives in primary healthcare institutions.

The incentives influencing employee behavior have been studied a lot. Appropriate organizational incentives such as a reasonable compensation system can effectively motivate employees to improve their satisfaction and reduce quitting behavior (34). Based on these abundant research, to further explore the association between incentives and quiet quitting, which is an emerging and hidden quiet quitting behavior. This study proposes the third hypothesis:

Hypothesis 3: Organizational incentives have a negative association with quiet quitting behavior of PHC workers.

Beyond its influence on formal structure, goal-setting also shapes the informal organizational environment. According to Goal-Setting Theory, when goals are clearly defined and perceived as attainable, they reduce role ambiguity and create shared performance expectations among employees (33, 41). Regular feedback on goal progress reinforces these shared understandings, embedding cooperative norms and mutual accountability into daily routines (33). Through these mechanisms, goal-setting cultivates a positive organizational culture characterized by trust, collaboration, and alignment with institutional mission. Organizational culture refers to organization's values, beliefs, shared symbols, and expectations of its members (35). The associations between organizational culture and goal-setting have been assumed in numerous qualitative research, in which some reveals that the effective goal-setting promotes positive cooperation culture in enterprises (36). Based on above research, this study proposes the fourth hypothesis:

Hypothesis 4: Goal setting has a positive association with organizational culture in primary healthcare institutions.

There exist sound evidences on relationship between organizational culture (such as hospital culture) and employee behavior. A systematic review showed that a positive organizational safety culture can reduce emotional exhaustion, burnout, and turnover intention among healthcare workers (37). And some studies revealed a positive organizational culture can also reduce turnover by increasing organization commitment (38). Based on above research, this study proposes the fifth hypothesis:

Hypothesis 5: Organizational culture has a negative association with quiet quit-ting behavior among PHC workers.

The famous Hawthorne experiment explores why production performance of employees remains at a moderate level and unable to be improved through centralized management supplemented by various incentives after managers have tried to set a variety of effective goals (39). This experiment demonstrates that organizational culture can also have an impact on employee working behavior, in addition to centralized management supplemented by various incentives measures that might impact employee working behavior (39). Based on this, the following hypothesis are proposed:

Hypothesis 6: Organizational centralization and organizational incentives have chain mediating effects between goal setting and quiet quitting behavior among PHC workers.

Hypothesis 7: Organizational culture has mediating effects between goal setting and quiet quitting behavior among PHC workers.

**Figure 1:**
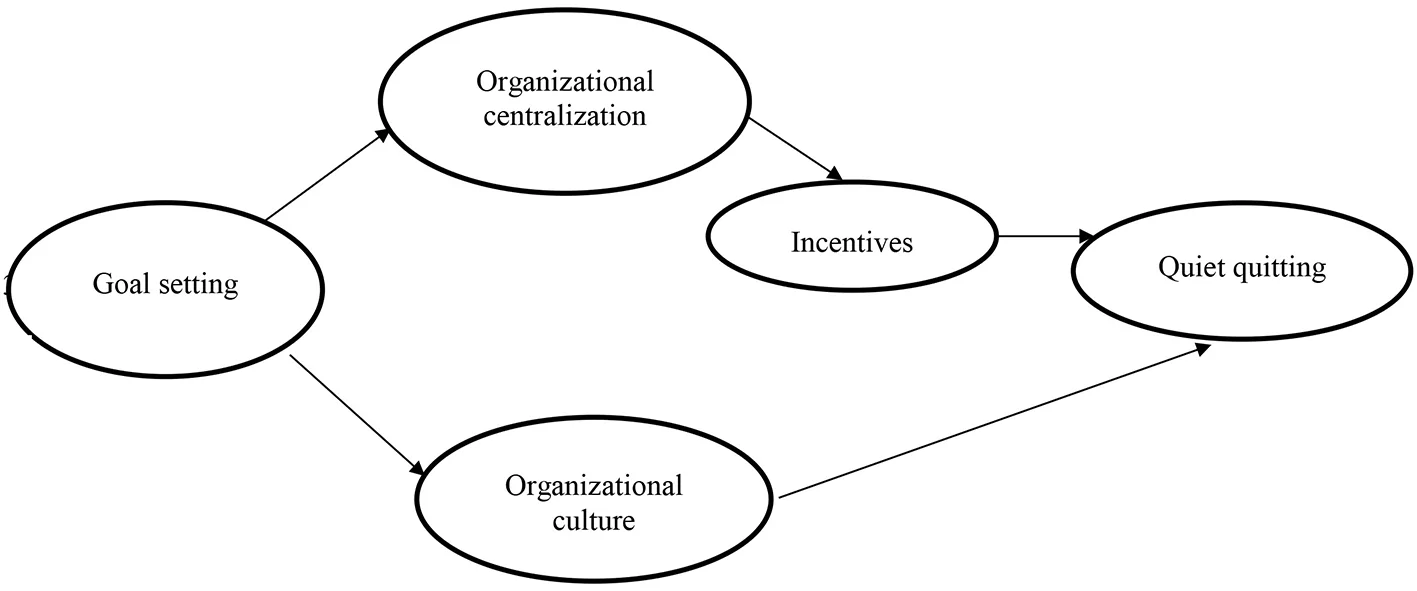
The theoretical model of the sequential-parallel mediation model.

Based on above hypotheses, our study constructs a sequential-parallel mediation with two parallel indirect pathways through which goal-setting affects quiet quitting among PHC workers. The first is a sequential-parallel mediation pathway (H1 → H2 → H3): appropriate goal-setting has a positive association with organizational centralization; organizational centralization has a positive association with organizational incentives; organizational incentives have a negative association with the quiet quitting of PHC workers. The second is a separate single mediation pathway (H4 → H5): specific goal-setting has a positive association with organizational culture, and positive organizational culture has a negative association with PHC workers' quiet quitting. By drawing theoretical analogies to conclusions from the Hawthorne Studies, the two proposed pathways share conceptual similarities with how formal and informal organizations operate. The concentration-incentive channel illustrates formal institutional safeguards, while the organizational culture channel encapsulates unwritten normative influences emerging within informal peer groups. The theoretical model in this study is shown in Figure 1.

## 3 Materials and methods

### 3.1 Variable measurements

The measurement of goal-setting was based on the Chinese version of Locke et al.'s questionnaires (40, 41), which has been previously applied in Chinese healthcare settings (42). Grounded in Locke and Latham's Goal-Setting Theory (43), which conceptualizes goal-setting as a multidimensional construct wherein these attributes do not operate in isolation but jointly reflect the overall quality of institutional goal setting level. To holistically reflect institutional goal setting level, we computed the average of the summed scores across the four subscales to generate a single composite indicator for the primary analyses. This aggregate indicator captures the overall quality of goal-setting within primary healthcare facilities and eliminates separate assessments of the effect of each individual dimension.

Organizational culture consists of 21 items covering seven dimensions as organizational coordination, communication, transformation, feedback, procedural fairness, competency development and organizational learning based on the Denison Organization Culture Scale modified (44), which has been validated in Chinese healthcare settings (45). Using the same composite-score approach as for goal-setting, we averaged the seven organizational culture subscales to form a single indicator that captures the overall cultural climate of primary healthcare facilities.

Organizational centralization is measured by three items, based on the organizational centralization scale modified (16, 46), which has been previously applied in Chinese healthcare settings (47).

Organizational incentives contain 13 items covering three dimensions of assessment incentive, revenue incentive and advancement incentive based on the Incentive mechanism for knowledge-based worker's scale (34, 48).

Quiet quitting contains nine items covering three dimensions of detachment, lack of initiative and lack of motivation based on the quiet quitting scale developed by Galanis et al. (7), which has been previously applied in Chinese primary health settings (42, 49).

Items are adapted by experts with acceptable reliability (Cronbach's α > 0.72) and validity (all *P*-value of Bartlett's Test of Sphericity less than 0.05). The factor structure is examined by validated factor analysis (CFA). Standardized factor loadings were examined to assess indicator reliability. Following the recommendation of Fornell and Larcker (50), all factor loadings exceeded the recommended threshold of 0.50, demonstrating adequate indicator reliability. All the Composite Reliability (C.R.) values for all constructs exceeded the recommended threshold of 0.70, indicating satisfactory internal consistency (51). Additionally, all Average Variance Extracted (AVE) values surpassed the acceptable criterion of 0.50 (50). These results collectively support the convergent validity of the measurement model. Responses were measured on a five-point Likert scale (1 = “extremely disagree” to 5 = “extremely agree”). The higher score means higher level of above variables. For example, higher the score indicating higher quiet quitting level.

Before launching the study, we pre-tested the instrument with 15 PHC workers to identify any problem linked to items, and to identify problems related to comprehension, flow, and duration of questionnaires. The pretest revealed no major problems linked to duration, structure, content, and flow.

### 3.2 Procedure and participants

This is a two-wave time-lagged observational study and 505 PHC workers were surveyed on-site in all primary healthcare institutions in a demonstrate county for poverty eradication of Hubei Province (Central China) in January and February 2026. The management ability of these institutions has been strengthened in the context of health poverty revitalization. The inclusion criteria were PHC workers such as general practitioners, nurses, medical technicians, public health physicians and other medical assistants, etc.; on duty; working year in primary healthcare institutions for ≥6 months; communicate without barriers to fill in the questionnaire voluntarily and independently. This study was conducted by means of a self-administered anonymous paper questionnaire, with written informed consent obtained following oral agreement on each questionnaire.

The detailed conducting procedure is as follows: The survey content of this study was developed based on the theoretical model established in Figure 1, which encompasses core research variables, we adopted a two-wave collection, splitting the measurement content across two distinct time points for separate data collection.

#### 3.2.1 Collection procedure

Time Point 1 (T1) Measurements (1st January 2026–7th January 2026): (1) measurement items for the four major management factors (goal setting, organizational centralization, organizational culture and incentives) of the primary healthcare institution; (2) demographic characteristics of primary healthcare workers (including gender, age, educational level, clinical working year, occupation, work income, professional title, social insurance, staffing of government affiliated institutions). Respondents were required to generate a personal matching code according to a predetermined rule to facilitate paired linkage of the time point 2 data.

Time Point 2 (T2) Measurements (29th January 2026–6th February 2026, administered 4 weeks after T1): contained only (3) measurement items for primary healthcare workers' quiet quitting. Respondents were again asked to enter their matching code, enabling longitudinal data pairing.

#### 3.2.2 Matching procedure for the two-time point data

To ensure accurate two-time point data linkage while strictly preserving anonymity, we adopted a “pre-coded reminder card with dual backup” strategy. The procedure was as follows:

(1) Pre-assignment of codes: Before the survey, project statisticians pre-assigned a unique random numeric code (e.g., A103, B207) to each participant, which was printed on separate dedicated reminder cards. (2) Distribution and instructions at T1: At the end of the T1 survey, field investigators distributed the reminder cards to participants and explicitly instructed them to: (1) keep the card with them at all times (e.g., tucked behind their work ID badge or placed in their wallet); and (2) take a photo of the card with their mobile phone for backup (optionally labeled as “survey code” for easy retrieval). (3) Effortless re-entry at T2: At T2 (4 weeks later), participants did not need to rely on memory. They simply retrieved the physical card or viewed the photo on their phones to accurately re-enter the same matching code.

All respondents participated on a voluntary basis. A standardized introductory statement was placed on the first page of the questionnaire, clearly indicating that this survey is supported by the National Natural Science Foundation of China, that the research results are used solely for academic analysis, that they do not involve personal performance evaluation, and that they are not incorporated into performance appraisals, in order to minimize social desirability bias.

#### 3.2.3 Data control quality

##### 3.2.3.1 Procedural control measures

Anonymous Matching and Ethical Safeguards: Data from the two waves are linked via personal matching codes, preventing the research team from tracing specific respondents' identities and ensuring anonymity. All data are aggregated for statistical analysis only, eliminating the risk of individual information disclosure.

Standardized Survey Administration Procedure: Prior to the formal survey, all level-based liaisons involved in questionnaire distribution received unified training to clarify the questionnaire explanation protocols, and guidelines for addressing frequently asked questions, ensuring consistency in survey implementation across all primary-level institutions. Questionnaires were primarily distributed in relatively concentrated work settings, such as departmental morning meetings and professional training sessions, to ensure that respondents had continuous and uninterrupted time for completion. Liaisons provided technical guidance only regarding completion methods without any intervention or direction on responses to specific items.

Temporal Separation and Variable Isolation: Core independent variables and the dependent variable were measured in two phases separated by a 4-week interval, effectively interrupting emotional consistency and immediate memory associations during responses, thereby fundamentally weakening the conditions conducive to common method bias. Attention Screening: Consistency check items (i.e., identical or highly similar items presented after a set number of intervening questions) were pre-set in the T1 questionnaire to identify careless respondents.

##### 3.2.3.2 Data cleaning criteria

Strict data cleaning was applied to questionnaires that were successfully collected and matched across both waves. The exclusion criteria are as follows:

(1) Excessively short response time (e.g., less than one-third of the average completion time, i.e., fewer than 3 min), considered indicative of careless responding;
(2) Obvious patterned responding (e.g., selecting the same option for all items or showing periodic oscillation patterns);
(3) Failure to pass the consistency check items (with clear contradictions between responses to identical items);
(4) Participation in only T1 or T2, rendering successful matching impossible.

### 3.3 Statistical analysis

All statistical analyses were performed using RStudio (Version 4.3.2; Posit Software, PBC, Boston, MA, USA) and AMOS (Version 24.0; IBM Corp., Armonk, NY, USA) for structural equation modeling (SEM) and bootstrap tests for mediating effects. The *P* < 0.05 was considered statistically significant.

## 4 Results

### 4.1 Demographic characteristics of participants

Table 1 presents demographic characteristics of study participants. There are 48.32% clinical physicians and nurses, 13.86% medical technicians, 22.97% public health physicians and 14.85% medical assistants. The average age of these PHC workers is around 39.15 ± 10.78, with around 15 average working years. The female PHC workers accounts for 63.76%. The proportion of bachelor's degree among PHC workers is 84.95%, of which the majority have primary title (48.12%). 91.09% of PHC workers are covered by health insurance and 56.24% of them are staffing of government affiliated institutions. The results of structural equation modeling (SEM) in this study are unchanged after considering demographic characteristics variables.

**Table 1:** Demographics characteristics of primary healthcare workers (*n* = 505).

Demographic	*N* (%)/mean ±SD
Gender	
Male	183 (36.24%)
Female	322 (63.76%)
Age	39.15 ± 10.78
Educational level	
Junior college and below	76 (15.05%)
University degree	429 (84.95%)
Master degree and above	0 (0%)
Clinical working year	15.28 ± 11.64
Occupation	
Clinical physician and nurse	244 (48.32%)
Medical technician	70 (13.86%)
Public health physician	116 (22.97%)
Medical assistance	75 (14.85%)
Work income	5.75 ± 2.49
Professional title	
No title	93 (18.41%)
Primary title	243 (48.12%)
Middle title	143 (28.32%)
Vice-senior title	24 (4.75%)
Senior title	2 (0.4%)
Social insurance	
Yes	460 (91.09%)
No	45 (8.91%)
Staffing of government affiliated institutions	
Yes	284 (56.24%)
No	221 (43.76%)

Data are expressed as mean ± SD or *n* (%). Work income is presented in ten thousand Chinese Yuan (CNY) per year; CNY, Chinese Yuan, 1 CNY ≈ 0.16US$.

### 4.2 Common-method deviation test

Common-method deviation test Harman's one-way test was used to test the collected data for common-method bias. The results extracted 11 factors with characteristic roots greater than one, of which the maximum factor variance explained only 32.11% (< 40%). This indicates no significant common method bias in this study.

### 4.3 Mean and correlation of goal-setting, organizational centralization, incentives, organizational culture and quiet quitting

The mean values with standard deviation of goal-setting and other variables are shown in Table 2. There is significant positive correlation among goal-setting, organizational centralization, incentives and organizational culture. The Spearman correlation coefficient revealed that above managerial factors were significantly negatively correlated with quiet quitting.

**Table 2:** Mean scores and correlation analysis of goal-setting, organizational centralization, organization culture, incentives and quiet quitting.

Variables	Mean	SD	Goal-setting	Organizational centralization	Organizational culture	Incentives	Quiet quitting
Goal-setting	3.99	0.39	**0.851**				
Organizational centralization	3.79	0.57	0.684^**^	**0.688**			
Organizational culture	4.03	0.43	0.475^**^	0.371^**^	**0.793**		
Incentives	3.84	0.45	0.461^**^	0.353^**^	0.497^**^	**0.708**	
Quiet quitting	2.11	0.59	−0.509^**^	−0.299^**^	−0.192^**^	−0.468^**^	**0.746**

Data are expressed as mean ± SD or n (%), ^**^P < 0.05. Diagonal bold values are the square roots of the average variance extracted (AVE).

### 4.4 Mechanism of goal-setting, organizational centralization, incentives, organizational culture on quiet quitting

**Figure 2:**
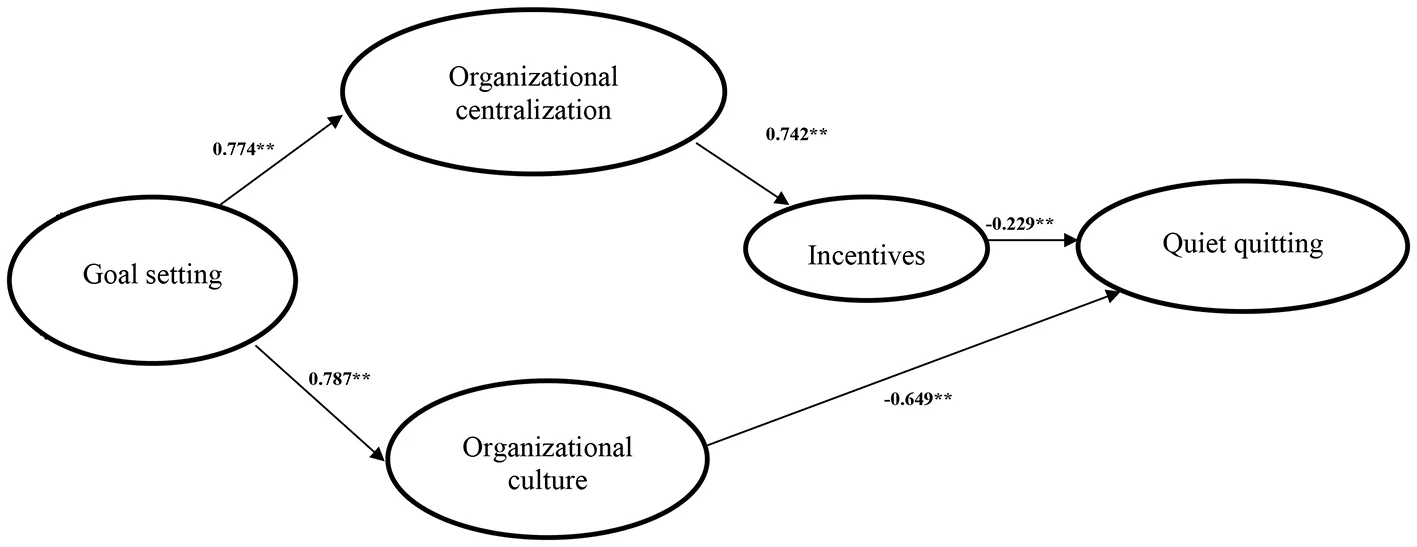
The sequential-parallel mediation model of multiple managerial factors and quiet quitting. **P < 0.05.

The model shows good fit with data root mean square error of approximation [RMSEA = 0.048 (excellent), standardized root mean square residual (SRMR) = 0.035 (excellent), incremental fit index (IFI) = 0.936 (excellent), comparative fit index (CFI) = 0.936 (excellent), tucker-lewis index (TLI) = 0.927 (excellent), χ2/df = 2.177 (excellent)]. As shown in Table 3 and Figure 2, the path coefficient of goal-setting on organizational centralization is 0.774 (*P* < 0.05), indicating that appropriate goal-setting has a significantly positive association with organizational centralization, with hypothesis 1 verified. The path coefficient of organizational centralization on incentives is 0.742 (*P* < 0.05), indicating that organizational centralization has a significantly positive association with incentives, with hypothesis 2 verified. The path coefficient of incentives on quiet quitting is −0.229 (*P* < 0.05), indicating incentives have a significantly negative association with quiet quitting, with hypothesis 3 verified. The above significant path coefficients indicate that organizational centralization and incentives play multiple chain mediating roles in the relationship between goal-setting and quiet quitting, with hypothesis 6 verified. The path coefficient of goal-setting on organizational culture is 0.787 (*P* < 0.05), indicating that appropriate goal-setting has a significantly positive association with organizational culture, with hypothesis 4 verified. The significant path coefficient of organizational culture on quiet quitting is −0.649 (*P* < 0.05), indicating that organizational culture has a significantly negative association with quiet quitting, with hypothesis 5 verified.

**Table 3:** Estimates of a sequential-parallel mediation model.

Path	Estimate	Standard error (S.E.)	Critical ratio (C.R.)	*P*-value
Goal-setting → organizational centralization	0.774	0.074	10.459	<0.05
Organizational centralization → incentives	0.742	0.325	2.283	<0.05
Incentives → quiet quitting	−0.229	0.072	−3.181	<0.05
Goal-setting → organizational culture	0.787	0.055	14.309	<0.05
Organizational culture → quiet quitting	−0.649	0.078	−8.321	<0.05
Goal-setting → quiet quitting	−0.127	0.147	−0.864	0.388

Meanwhile, paths of mechanism indicates that goal-setting shows a negative indirect association with quiet quitting through two parallel paths of organizational centralization, incentives and organizational culture separately, indicating that organizational centralization, incentives and organizational culture form two parallel mediator paths between goal-setting and quiet quitting. In further model exploration process, it was found that the path coefficient of goal-setting directly was not significantly associated with quiet quitting is −0.127 (*P* = 0.388), which indicated that goal-setting is not significantly and directly associated with quiet quitting behaviors. Therefore, the mediating effect in this study was considered as a completing mediating effect.

### 4.5 Specific mediation effects

The results of the mediation effect analysis of three mediators (organizational centralization, incentives and organizational culture) can be seen in Table 4. The effect values and confidence intervals for organizational centralization and incentives was estimated [indirect effect = −0.132, 95%CI = (−0.200, −0.051)], and the upper and lower Bootstrap 95 % confidence intervals did not contain 0. The results showed that the chain mediating effects of organizational centralization and incentives between goal-setting and quiet quitting were significant, with hypothesis 6 verified. In addition, the mediating effect of organizational culture between goal-setting and quiet quitting is also verified significant [indirect effect = −0.511, CI = (−0.631, −0.451)], with hypothesis 7 verified.

**Table 4:** Specific indirect effects and comparison (phantom modeling approach).

Outcome variable	Indirect effect through organizational centralization and incentives			Indirect effect through organizational culture			Contrast		
	Estimate	BCCI		Estimate	BCCI		Estimate	BCCI	
		Lower	Upper		Lower	Upper		Lower	Upper
Quiet quitting	−0.132^**^	−0.200	−0.051	−0.511^**^	−0.631	−0.451	0.379^**^	0.252	0.574

BCCI, Bias-corrected confidence intervals. ^**^P < 0.05.

Specific effect testing is of focal interest in multiple chain mediation approaches. They permit comparisons and rankings of the mediators which provide a better understanding of mediation processes. In our case, the association between goal-setting and quiet quitting or work effectiveness is carried through two parallel mediating paths (via organizational centralization and incentives vs. organizational culture). Therefore, it is of interest to know which mediated effect is stronger and more relevant (52). To further test our mediation effects, we conducted a bootstrap analysis with AMOS, iterating 5,000 times. The results are also given in Table 4, with comparative results of specific effects. According to the significant difference in effect of two paths (difference = 0.379, *P* < 0.01), the path effect of organizational culture (indirect effect = −0.511, *P* < 0.01) is significantly stronger than path of combined effect of organizational centralization and incentives (indirect effect = −0.132, *P* < 0.01). This suggests that for PHC workers, the negative association between organizational culture and their quiet quitting behavior is much stronger than that of joint association of organizational centralization and incentives.

## 5 Discussion

Since there is currently no universally accepted or China-specific cutoff point for identifying quiet quitting among primary healthcare workers, we classified participants based on the cutoff value (score > 2.06) established by Galanis et al. in their Greek healthcare sample (7, 53). Using this criterion, approximately 47% of our respondents were identified as quiet quitters. However, as this cutoff was derived from a different cultural and occupational context and has not been validated among the domestic primary healthcare workforce, the prevalence rate we observed should be interpreted as an exploratory estimate. Nevertheless, even as a preliminary finding, this figure suggests that quiet quitting is to some extent prevalent among primary healthcare workers.

This study is the first to quantitatively explore the indirect pathways through which multiple managerial factors are associated with quiet quitting. First, a quantitative model was developed to examine how goal-setting is associated with organizational centralization in primary healthcare institutions (β = 0.774, *P* < 0.05), which provides quantitative evidence for the view that goal-setting is associated with organizational centralization (43, 54). The goals of supplying high-quality of medical service requires high level of centralization of medical institutions. The significance of this goal to form a high centralized medical institution is that, in such highly centralized system managers can easily control and coordinate work duties of PHC workers to achieve one big goal such as one health in all, especially in areas with poor managerial functions (55). Secondly, our research showed that incentives in primary healthcare institutions might be greatly positively associated with by centralization (β = 0.742, *P* < 0.05). This result is consistent with the findings of healthcare management studies and extends the research scenarios, which demonstrate that centralized organizational structures in healthcare settings are associated with greater reliance on formal HR practices and external incentives to enhance employee motivation and reduce turnover intent. The underlying mechanism is that centralization limits employee discretion and participation in decision making, thereby weakening their psychological empowerment, which in turn necessitates more tangible rewards to sustain work engagement (56).

On the other hand, another quantitative research argues that the higher degree of centralization, the less likely for financial institutions to use the compensation incentive to motivate executives enthusiasm (β = −0.134, *P* < 0.05) (57). We discovered that centralization had a positive association with incentives of primary healthcare institutions when compared to its opposite effect direction. This finding may be due to different subjects and broadly defined incentives in this study. Thirdly, our study found that incentives are significantly negatively associated with quiet quitting among PHC workers (β = −0.229, *P* < 0.05). This result indicates that one feasible measure for incentives at work is to give PHC workers priority in job title evaluations, which encourages them to work diligently at primary healthcare institutions (34, 58). This is also consistent with findings of many previous studies exploring employees' working behavior (34, 58). For instance, a research in a back office of a Belgian financial institution demonstrates that appropriate incentives such as satisfied compensation system in this institution can curb employees' quitting intention (β = −0.50, *P* < 0.001) (59). Appropriate incentives, including performance appraisal system, can lower nurses' intention to quitting (β = −0.159, *P* < 0.001), according to another research in Chinses medical system (48). These studies support our findings in general, although they vary from us in terms of the extent to which incentives have an association with quitting-related research. The variation in influencing degree may due to our different study subjects and broadly defined incentives that we also consider other kinds of incentives like advancement, not only performance appraisal system. Fourthly, this study suggests that appropriate goal-setting is significantly negatively associated with positive organizational culture, which riches related evidence of idea or hypothesis in previous studies that goals in the organization can shape their culture (60). Fifthly, our study suggests that positive organizational culture, such as providing good learning atmosphere, is significantly negatively associated with quiet quitting of PHC workers (β = −0.649, *P* < 0.05).

Consistent with findings of previous studies in exploring factors of employee's quitting behavior, which suggested that positive and good organization-employee relations are negatively related to employees' quitting intention (61). Moreover, A cross-sectional study in Korea shows that consensual culture has negative effect on nurse's quitting intention (β = −0.118, *P* < 0.01) (38). In comparison, we found a slightly stronger effect of organizational culture on quiet quitting among PHC workers in this study, which may be due to our broadly defined organizational culture and different subjects.

It is noticeable that our mechanism forms two parallel paths between goal-setting and quiet quitting. The one path is that goal-setting shows an indirect association with quiet quitting through organizational centralization and incentives. Another path is that goal-setting shows an indirect association with quiet quitting behavior through organizational culture. It is worth noticing that the association between organizational culture and quiet quitting [indirect effect = −0.511, 95% CI = (−0.631, −0.451)] was much stronger than the joint mediation associations between organizational centralization and incentives [indirect effect = −0.132, 95% CI = (−0.200, −0.051)]. This result is consistent with resonates with the core conclusions of Hawthorne studies by Elton Mayo conducted in western electric company, which highlighted the vital influence of social and psychological forces on workers' behavioral patterns (62). The Hawthorne studies famously indicated that aside from formal incentives, employees' emotional demands including a sense of security, belonging and recognition exert crucial impacts on their work performance and engagement (62). However, traditional management emphasized centralized control supplemented by incentives to achieve goal of improving performance, often overlooking the informal and humanistic aspects of work, which is similar to current views in medical management that regarding unreasonable remuneration system as the core factor which have negative influence on physician's working behavior, and paying insufficient attention to physicians' emotional comforts (62).

Nevertheless, this finding should be interpreted with caution given our single-county sampling frame. The strong association observed for organizational culture may reflect the specific policy context of this demonstration county, where intensive investments in management capacity and workforce development have been made under the health poverty revitalization policy. Whether this pattern holds in other settings requires further investigation. Our findings highlight the need for managers to place greater emphasis on fostering positive organizational culture, even within contexts with equitable remuneration and sound hardware resources. Our results indicate that both the incentive pathway and cultural pathway are significantly linked to quiet quitting, yet the cultural pathway demonstrates a markedly stronger correlation. This does not mean incentive systems are negligible; our data verify that incentives remain significantly and negatively associated with quiet quitting. Instead, organizational culture serves as a more impactful tool to mitigate employee disengagement. Accordingly, incentives and organizational culture should not be treated as mutually exclusive alternatives. Managers ought to pursue a balanced governance strategy: sustaining robust incentive mechanisms, alongside continuous efforts to cultivate supportive organizational culture.

## 6 Conclusions and limitation

This study provides valuable insights into quiet quitting among PHC workers under perspectives of multiple managerial factors. By exploring research subjects from a demonstrate county whose management capacity has been enhanced under health poverty revitalization policy, a mechanism with two parallel pathways is constructed to explain that well-designed goal-setting is indirectly associated with quiet quitting through organizational centralization and incentives vs. organizational culture, separately. Importantly, organizational culture shows a much stronger negative association with quiet quitting than the combined effect of organizational centralization and incentives. These findings suggest that while both centralization-incentive and cultural mechanisms are relevant, organizational culture exhibits a stronger association with quiet quitting. This implies that managers in the healthcare field may benefit from prioritizing the development of a positive organizational culture for PHC workers, which is a key element of a healing workplace, alongside maintaining effective incentive systems, rather than relying on either mechanism in isolation. Based on these findings, a more forward-looking management concept for healthcare settings is proposed. Managers should prioritize building a positive organizational culture for PHC workers, a key element of a healing workplace, rather than relying primarily on incentives and centralized control to achieve high-quality development of medical services. Such a balanced approach may be associated with lower quiet quitting and improved employee wellbeing and organizational performance.

This study has the following limitations. Firstly, the single county sampling limits the generalizability of our findings. The specific organizational and socioeconomic context of this demonstration county may not be representative of other primary healthcare settings because our data came from a single Chinese county. Therefore, multi-site studies are needed to validate the external validity of our results. Secondly, this cross-sectional self-reported data cannot draw causal conclusions and risks subjective response biases. Future longitudinal or experimental research with objective measurements is required to confirm causality. Furthermore, this study only analyzed aggregated goal-setting scores, and subsequent studies may unpack its four subdimensions to explore differentiated mechanisms. Thirdly, although our findings resonate with the Hawthorne studies, we did not directly measure its core constructs. These interpretations are therefore exploratory and should be read with caution. Future research with direct measures is needed to verify these mechanisms.

## Data Availability

The datasets presented in this article are not readily available because data may be accessed via reasonable requests approved by the corresponding author (Qianqian Xu). Restrictions apply to data sharing, as the dataset was used under exclusive license for this study and cannot be released publicly. Requests to access the datasets should be directed to qq_xu93@hust.edu.cn.

## References

[1] Assefa Y Tesfaye D Damme WV Hill PS. Effectiveness and sustainability of a diagonal investment approach to strengthen the primary health-care system in Ethiopia. Lancet. (2018) 392:1473–81. doi: 10.1016/S0140-6736(18)32215-3 30343861

[2] Maglalang DD Sorensen G Hopcia K Hashimoto DM Katigbak C Pandey S . Job and family demands and burnout among healthcare workers: the moderating role of workplace flexibility. SSM Popul Health (2021) 14:100802. doi: 10.1016/j.ssmph.2021.100802 33997249 PMC8102798

[3] Shen K McGarry BE Gandhi AD. Health care staff turnover and quality of care at nursing homes. JAMA Intern Med. (2023) 183:1247–54. doi: 10.1001/jamainternmed.2023.5225 37812410 PMC10562988

[4] Boy Y Sürmeli M. Quiet quitting: a significant risk for global healthcare. J Glob Health (2023) 13:03014. doi: 10.7189/jogh.13.03014 36995298 PMC10062397

[5] Forrester N. Fed up and burnt out: “quiet quitting” hits academia. Nature (2023) 615:751–3. doi: 10.1038/d41586-023-00633-w 36869111

[6] Hamouche S Koritos C Papastathopoulos A. Quiet quitting: relationship with other concepts and implications for tourism and hospitality. Int J Contemp Hosp Manag. (2023) 35:4297–312. doi: 10.1108/IJCHM-11-2022-1362

[7] Galanis P Moisoglou I Malliarou M Papathanasiou IV Katsiroumpa A Vraka I . Quiet quitting among nurses increases their turnover intention: evidence from Greece in the post-COVID-19 era. Healthcare (2024) 12:79. doi: 10.3390/healthcare12010079 PMC10779139 38200985

[8] Kumar S. Quiet quitting and its relevance to the medical profession. MGM J Med Sci. (2023) 10:1. doi: 10.4103/mgmj.mgmj_42_23

[9] Wallace JE Lemaire JB Ghali WA. Physician wellness: a missing quality indicator. Lancet. (2009) 374:1714–21. doi: 10.1016/S0140-6736(09)61424-0 19914516

[10] Heins E Parry R. The role of wage bargaining partners in public sector reform: the case of primary care contracts. Eur J Ind Relat. (2011) 17:381–96. doi: 10.1177/0959680111420722

[11] Xu J Shi Y Cheng F Liang W. China's public health system: time for improvement. Lancet Public Health (2021) 6:e869–70. doi: 10.1016/S2468-2667(21)00250-4 34838191 PMC8616565

[12] Mabuchi S Alonge O Tsugawa Y Bennett S. Measuring management practices in primary health care facilities - development and validation of management practices scorecard in Nigeria. Glob Health Action (2020) 13:1763078. doi: 10.1080/16549716.2020.1763078 32508273 PMC7448912

[13] Ward C Phiri ERM Goodman C Nyondo-Mipando AL Malata M Manda WC . What is the relationship between hospital management practices and quality of care? A systematic review of the global evidence. Health Policy Plan. (2025) 40:409–21. doi: 10.1093/heapol/czae112 39575503 PMC11886796

[14] Zhu Y Guo R Dou L Zhao Y Li S Qiao Y . Development and evaluation of a hospital management practice rating scale. J Hosp Adm. (2018) 7:9. doi: 10.5430/jha.v7n1p929736199

[15] Tsai TC Jha AK Gawande AA Huckman RS Bloom N Sadun R. Hospital board and management practices are strongly related to hospital performance on clinical quality metrics. Health Aff. (2015) 34:1304–11. doi: 10.1377/hlthaff.2014.1282 26240243

[16] Schminke M Cropanzano R Rupp DE. Organization structure and fairness perceptions: the moderating effects of organizational level. Organ Behav Hum Decis Process. (2002) 89:881–905. doi: 10.1016/S0749-5978(02)00034-1

[17] Aloisio LD Gifford WA McGilton KS Lalonde M Estabrooks CA Squires JE. Individual and organizational predictors of allied healthcare providers' job satisfaction in residential long-term care. BMC Health Serv Res. (2018) 18:491. doi: 10.1186/s12913-018-3307-3 29940949 PMC6019323

[18] Child J. Managerial and organizational factors associated with company performance part I. J Manag Stud. (1974) 11:175–89. doi: 10.1111/j.1467-6486.1974.tb00693.x

[19] Rooney JA Gottlieb BH Newby-Clark IR. How support-related managerial behaviors influence employees: an integrated model. J Manag Psychol. (2009) 24:410–27. doi: 10.1108/02683940910959744

[20] Fan L Yang J Hou Q Feng S Chen A Han L. Impact of job resources on organizational citizenship behavior among primary care staff in Guangzhou, China: a cross-sectional study. Front Public Health. (2025) 13:1580148. doi: 10.3389/fpubh.2025.1580148 40620565 PMC12226538

[21] Li X Yang C Liu L Ding Y Xue J He J . Configurational paths to turnover intention among primary public health workers in Liaoning province, China: a fuzzy-set qualitative comparative analysis. BMC Public Health (2024) 24:369. doi: 10.1186/s12889-024-17881-8 38317139 PMC10840158

[22] Zhao R Zhang J Li M Loban E Nicolas S Martiland E . Primary care physicians' work conditions and their confidence in managing multimorbidity: a quantitative analysis using job demands–resources model. Fam Pract. (2024) 41:977–84. doi: 10.1093/fampra/cmad099 37851711

[23] Bloom N Van Reenen J. Measuring and explaining management practices across firms and countries^*^. Q J Econ. (2007) 122:1351–408. doi: 10.1162/qjec.2007.122.4.1351

[24] McConnell KJ Hoffman KA Quanbeck A McCarty D. Management practices in substance abuse treatment programs. J Subst Abuse Treat. (2009) 37:79–89. doi: 10.1016/j.jsat.2008.11.002 19195813 PMC2755538

[25] He Q Liu GG Chen J Yuan L Hong X Zhang Z. How does management matter for hospital performance? Evidence from the global hospital management survey in China. Int J Health Policy Manag. (2024) 13:8478. doi: 10.34172/ijhpm.8478 39624864 PMC11806224

[26] Li J Zhao T Liu Z Chen L Wang X Pan J. Health poverty reduction policies and effectiveness evaluation in China since 2015: a narrative review. Inq J Health Care Organ Provis Financ. (2025) 62:00469580251337232. doi: 10.1177/00469580251337232 40376926 PMC12084708

[27] van de Klundert J (Joris) De Korne D Yuan S Wang F Van Wijngaarden J. “Hybrid” top down bottom up health system innovation in rural China: a qualitative analysis. PLoS ONE (2020) 1:e0239307. doi: 10.1371/journal.pone.0239307 33027287 PMC7540887

[28] Ni Y Wang Y Wen Z Fang J Xu J Wu S . Optimization path of primary public health service talent team construction: a largescale survey in Huaihai economic zone, China. Front Public Health (2024) 12:1399857. doi: 10.3389/fpubh.2024.1399857 39234097 PMC11371681

[29] Koskosas IV Charitoudi G Louta M. The role of organizational cultures in information-systems security management: a goal-setting perspective. J Leadersh Stud. (2008) 2:7–17. doi: 10.1002/jls.20042

[30] Khatri RB Endalamaw A Erku D Wolka E Nigatu F Zewdie A . Contribution of health system governance in delivering primary health care services for universal health coverage: a scoping review. PLoS ONE (2025) 20:e0318244. doi: 10.1371/journal.pone.0318244 40019911 PMC11870385

[31] Lebourges M Masclet D. Centralized vs. decentralized incentives in teams: experimental evidence. Revue économique. (2024) 75:975–1042. doi: 10.3917/reco.755.0975

[32] Bonenberger M Aikins M Akweongo P Wyss K. The effects of health worker motivation and job satisfaction on turnover intention in Ghana: a cross-sectional study. Hum Resour Health (2014) 12:43. doi: 10.1186/1478-4491-12-43 25106497 PMC4130118

[33] Lin N. Capitalism in China: a centrally managed capitalism (CMC) and its future. Manag Organ Rev. (2011) 7:63–96. doi: 10.1111/j.1740-8784.2010.00203.x

[34] Sang L Zheng X Chen G Bai Z Chen R. The effect of incentive factors on turnover intention of PHC workforce in rural China—from the perspective of two-factor theory. BMC Health Serv Res. (2024) 24:1486. doi: 10.1186/s12913-024-11790-7 39604900 PMC11603648

[35] Panda DK. Impact of organizational culture on strategic planning. Manag Decis. (2021) 60:1349–68. doi: 10.1108/MD-10-2020-1375

[36] Kaul A. Culture vs strategy: which to precede, which to align? J Strategy Manag. (2019) 12:116–36. doi: 10.1108/JSMA-04-2018-0036

[37] Finn M Walsh A Rafter N O'Connor P Lydon S Kerin MJ . Effect of interventions to improve safety culture on healthcare workers in hospital settings: a systematic review of the international literature. BMJ Open Qual. (2024) 13:e002506. doi: 10.1136/bmjoq-2023-002506 38719514 PMC11086522

[38] San PJ Hyun KT. Do types of organizational culture matter in nurse job satisfaction and turnover intention? Leadersh Health Serv. (2009) 22:20–38. doi: 10.1108/17511870910928001

[39] Bagni G Marçola JA Escrivão Filho E Nagano MS Philippsen L Jr. Designing a hybrid organization structure based on competences: the importance of the organizational structure. Bus Process Manag J. (2025) 31:2849–70. doi: 10.1108/BPMJ-02-2024-0098

[40] Klein HJ Wesson MJ Hollenbeck JR Wright PM DeShon RP. The assessment of goal commitment: a measurement model meta-analysis. Organ Behav Hum Decis Process. (2001) 85:32–55. doi: 10.1006/obhd.2000.2931 11341816

[41] Locke EA Frederick E Lee C Bobko P. Effect of self-efficacy, goals, and task strategies on task performance. J Appl Psychol. (1984) 69:241–51. doi: 10.1037/0021-9010.69.2.241

[42] Hu J Zou D Xu Q Wu Y Fan S Wang Y Zhang X. Impact of work goals on quiet quitting among chinese primary health professionals based on goal setting theory: a cross-sectional survey. Healthcare. (2025) 13:2739. doi: 10.3390/healthcare13212739 41228106 PMC12609706

[43] Smith KG Locke EA Barry D. Goal setting, planning, and organizational performance: an experimental simulation. Organ Behav Hum Decis Process. (1990) 46:118–34. doi: 10.1016/0749-5978(90)90025-5

[44] Ashkanasy NM Broadfood LE Falkus S. Questionnaire Measures or Organizational Culture. In: Ashkanasy N Wilderom CPM Peterson MF, editors. Handbook of Organizational Culture and Climate. Thousand Oaks, CA: Sage Publications (2000). p. 131–45.

[45] Ping Z Lan LI Zhi-liu T Jia-yan H Di XUE. Research on a tool for organizational culture assessment of public general hospitals in China. Chin J Hosp Adm. (2011) 27:433–6.

[46] Shi J Sun X Meng K. Identifying organisational capability of hospitals amid the new healthcare reform in China: a Delphi study. BMJ Open (2021) 11:e042447. doi: 10.1136/bmjopen-2020-042447 33408207 PMC7789467

[47] Zhu L Peng Z Liu L Ling S. Combining resource, structure and institutional environment: a configurational approach to the mode selection of the integrated healthcare in county. Int J Environ Res Public Health (2019) 16:2975. doi: 10.3390/ijerph16162975 31430889 PMC6719034

[48] Wang P Lu Z Sun J. Influential effects of intrinsic-extrinsic incentive factors on management performance in new energy enterprises. Int J Environ Res Public Health (2018) 15:292. doi: 10.3390/ijerph15020292 29419730 PMC5858361

[49] Zhang D Zheng H Zeng Y Yu X Zhao Y Gan Y . Psychometric properties of the Chinese version of the quiet quitting scale. BMC Nurs. (2025) 24:270. doi: 10.1186/s12912-025-02921-4 40069793 PMC11900426

[50] Fornell C Larcker DF. Evaluating structural equation models with unobservable variables and measurement error. J Mark Res. (1981) 18:39–50. doi: 10.1177/002224378101800104

[51] Bagozzi RP Yi Y. On the evaluation of structural equation models. J Acad Mark Sci. (1988) 16:74–94. doi: 10.1007/BF02723327

[52] Rose JS Chassin L Presson CC Sherman SJ eds. Multivariate Applications in Substance Use Research: New Methods for New Questions, 1st edn. Hove: Psychology Press. (2000). doi: 10.4324/9781410604217

[53] Galanis P Katsiroumpa A Vraka I Siskou O Konstantakopoulou O Moisoglou I . Quiet quitting among employees: a proposed cut-off score for the “Quiet Quitting” Scale. Arch Hellen Med. (2024) 41:381–7.

[54] Chandler AD Jr. Strategy and Structure: Chapters in the History of the Industrial Enterprise. Oxford: MIT Press (1962).

[55] Li H Liu K Gu J Zhang Y Qiao Y Sun X. The development and impact of primary health care in China from 1949 to 2015: a focused review. Int J Health Plann Manage. (2017) 32:339–50. doi: 10.1002/hpm.2435 28670754

[56] Kim J Wehbi N DelliFraine JL Brannon D. The joint relationship between organizational design factors and HR practice factors on direct care workers' job satisfaction and turnover intent. Health Care Manage Rev. (2014) 39:174–84. doi: 10.1097/HMR.0b013e31828c8b8f 23558755

[57] Sharma B Fayyaz A. The effect of hegemonic power on executive compensation. Int J Commer Manag. (2000) 10:79–91. doi: 10.1108/eb047411

[58] Li J Qin X Wang H Yuan B. Performance-based payments and intrinsic motivation among primary healthcare workers in China: a cross-sectional survey. BMJ Open (2025) 15:e090145. doi: 10.1136/bmjopen-2024-090145 40562553 PMC12198835

[59] De Gieter S Hofmans J. How reward satisfaction affects employees' turnover intentions and performance: an individual differences approach. Hum Resour Manag J. (2015) 25:200–16. doi: 10.1111/1748-8583.12072

[60] Chadwick IC Raver JL. Motivating organizations to learn: goal orientation and its influence on organizational learning. J Manag. (2015) 41:957–86. doi: 10.1177/0149206312443558

[61] Mohr DC Burgess JF Young GJ. The influence of teamwork culture on physician and nurse resignation rates in hospitals. Health Serv Manage Res. (2008) 21:23–31. doi: 10.1258/hsmr.2007.007011 18275662

[62] Franke RH. The Hawthorne experiments: re-view. Am Sociol Rev. (1979) 44:861–7. doi: 10.2307/2094534

