# Supplementary material for: Workplace management practices and quiet quitting among primary healthcare workers: a sequential-parallel mediation analysis
Source: Front Public Health. 2026 Aug 6;14:1903586. doi: 10.3389/fpubh.2026.1903586 (PMC13502071; doi:10.3389/fpubh.2026.1903586)
Supplement: Supplementary file 1 [file Supplementary_file_1.docx]

Supplementary Material

**1.Appendix A**

**Table S1. Confirmatory Factor Analysis Results (Goal-setting Dimension)**

| Dimensions | Items | Factor loadings | Composite Reliability (C.R.) | AVE |
| --- | --- | --- | --- | --- |
| Goal clarity | GL1 | 0.877 | 0.963 | 0.811 |
|  | GL2 | 0.926 |  |  |
|  | GL3 | 0.917 |  |  |
|  | GL4 | 0.919 |  |  |
|  | GL5 | 0.858 |  |  |
|  | GL6 | 0.908 |  |  |
|  | GL7 | 0.898 |  |  |
| Goal attainment | GA1 | 0.864 | 0.929 | 0.777 |
|  | GA2 | 0.906 |  |  |
|  | GA3 | 0.885 |  |  |
|  | GA4 | 0.866 |  |  |
|  | GA5 | 0.886 |  |  |
| goal identity | GI1 | 0.826 | 0.944 | 0.771 |
|  | GI2 | 0.895 |  |  |
|  | GI3 | 0.909 |  |  |
|  | GI4 | 0.911 |  |  |
|  | GI5 | 0.844 |  |  |
| goal commitment | GC1 | 0.716 | 0.870 | 0.587 |
|  | GC2 | 0.687 |  |  |
|  | GC3 | 0.584 |  |  |
|  | GC4 | 0.885 |  |  |
|  | GC5 | 0.884 |  |  |

**Table S2. Confirmatory Factor Analysis Results (Organizational culture Dimension)**

| **Variable** | **Items** | **Factor loadings** | **Composite Reliabilty (C.R.)** | **AVE** |
| --- | --- | --- | --- | --- |
| organizational coordination | COO1 | 0.615 | 0.711 | 0.585 |
|  | COO2 | 0.861 |  |  |
|  | COO3 | 0.798 |  |  |
| organizational learning | OL1 | 0.863 | 0.895 | 0.741 |
|  | OL2 | 0.863 |  |  |
|  | OL3 | 0.855 |  |  |
| organizational communication | OCO1 | 0.897 | 0.912 | 0.851 |
|  | OCO2 | 0.941 |  |  |
|  | OCO3 | 0.928 |  |  |
| organizational change | OC1 | 0.891 | 0.881 | 0.806 |
|  | OC2  OC3 | 0.915  0.887 |  |  |
| organizational feedback | OF1 | 0.789 | 0.814 | 0.698 |
|  | OF2 | 0.891 |  |  |
|  | OF3 | 0.823 |  |  |
| procedural fairness | PF1  PF1  PF2 | 0.924  0.940  0.910 | 0.915 | 0.855 |
| competency development | LD2 | 0.854 | 0.823 | 0.726 |
|  | LD3 | 0.789 |  |  |
|  | LD4 | 0.908 |  |  |

**Table S3. Confirmatory Factor Analysis Results (Initiative Dimension)**

| Dimensions | Items | Factor loadings | Composite Reliability (C.R.) | AVE |
| --- | --- | --- | --- | --- |
| revenue incentive | RI1  RI2  RI3 | 0.843  0.814  0.799 | 0.859 | 0.671 |
| assessment incentive | AS1  AS2  AS3  AS4  AS5 | 0.771  0.663  0.841  0.741  0.826 | 0.879 | 0.594 |
| advancement incentive | AD1  AD2  AD3  AD4  AD5 | 0.857  0.888  0.910  0.872  0.631 | 0.921 | 0.702 |

**Table S4. Confirmatory Factor Analysis Results (Quiet quitting Dimension)**

| Dimensions | Items | Factor loadings | Composite Reliability (C.R.) | AVE |
| --- | --- | --- | --- | --- |
| Detachment | DE1  DE2  DE3  DE4 | 0.601  0.611  0.661  0.879 | 0.758 | 0.611 |
| Lack of initiative | LI1  LI2  LI3 | 0.807  0.897  0.843 | 0.886 | 0.722 |
| Lack of motivation | LM1  LM2 | 0.928  0.922 | 0.922 | 0.856 |

**Table S5. Confirmatory Factor Analysis Results (Organizational centralization Dimension)**

| Dimensions | Items | Factor loadings | Composite Reliability (C.R.) | AVE |
| --- | --- | --- | --- | --- |
| Organizational centralization | OC1 | 0.699 | 0.776 | 0.688 |
|  | OC2 | 0.781 |  |  |
|  | OC3 | 0.711 |  |  |

**2.Appendix B**

**Expert Consultation Method**

**Composition of the Expert Panel**

We assembled an expert panel consisting of eight participants with the following backgrounds:

(1) Two senior administrators of primary healthcare institutions (directors of community health service centers);

(2) Three experienced general practitioners, each with more than 15 years of frontline clinical practice in primary care;

(3) Three university professors specializing in health policy and hospital management.

This multidisciplinary panel design balances the practical operational realities of grassroots medical facilities with rigorous academic standards.

**Consultation Procedures**

Two rounds of semi-structured group discussions were held with the expert panel.

**Round 1: Open Classification**

All core items covering the five dimensions of the original World Management Survey (WMS) were extracted. Taking the unique characteristics of primary care into account — decentralized decision-making, substantial professional autonomy of medical staff, team-based work orientation, and performance-driven incentives — each expert independently assigned every item to one of the newly proposed constructs or recommended its removal.

**Round 2: Consensus Reaching**

The research team compiled all items with inconsistent classifications from the first round and hosted a facilitated group discussion, followed by formal voting. An item was retained and reclassified only if it achieved a consensus rate of no less than 85% among experts.

**Theoretical Foundation**

Throughout the expert consultation process, management process theory (planning, organizing, leading, controlling) served as our analytical framework. This ensured that the newly developed dimensions logically and comprehensively capture core managerial functions within primary care contexts. Following the above procedures, the original WMS dimensions were restructured into four managerial factors: Goal-Setting, Organizational Centralization, Organizational Culture, and Incentives.

To fully document the whole adaptation procedure and guarantee transparency and traceability, a detailed mapping table (Table 1) is presented below. It specifies how each dimension and its core items from the original WMS were revised, alongside the expert panel’s rationales for every reclassification, integration, and item deletion.

**Table S6. Mapping of Original WMS Domains to Adapted Managerial Constructs**

| Original HMP/WMS Dimension | Core Content of Dimension | Adapted Construct | Mapping Rationale | Content Retained / Deleted in Our Survey |
| --- | --- | --- | --- | --- |
| **Target Management** | Target balance; target setting; target sharing; target stretch | **Goal-Setting** | Directly retained and conceptually expanded with Locke‘s Goal-Setting Theory. The HMP target management dimension provides foundational items on target clarity, consistency, and awareness; we supplemented additional items on goal clarity, attainability, identity, and commitment from Locke’s theory to more comprehensively capture the full theoretical construct. | **Retained:** All four subdimensions (balance, setting, sharing, stretch).  **Supplemented:** Additional goal-setting theory items.  **Deleted:** None. |
| **Operations Management** | Medical environment; service layout; patient-centered services; clinical pathway; continuous quality improvement | **Organizational Culture**  (conceptually refocused) | In primary care, the value of operational management lies not in physical infrastructure compliance but in whether teams use quality improvement, patient-centered processes, and clinical standardization for collaborative learning. These retained elements map directly onto the **learning & development** and **coordination & communication** subdimensions of the Denison Organizational Culture Model, which underpins our measurement. | **Retained:** Quality improvement, patient-centered services, and clinical pathway items.  **Deleted:** Infrastructure items (space layout, distance measures, signage). |
| **Performance Management** | Hospital/department/staff performance appraisal; penalties; workload assessment | **Organizational Centralization** / **Organizational Culture** /  **Incentives**  *(disaggregated)* | This dimension was disaggregated across three adapted constructs based on their distinct theoretical functions: (1) Following Aiken and Hage's conceptualization of centralization as hierarchical concentration of decision-making authority, we extracted approval-requirement components; (2) review/feedback mechanisms were retained to capture learning culture; (3) reward/penalty components were extracted to capture incentives. | **Retained—Centralization:** Decision-making authority.  **Retained—Culture:** Review, dialogue, feedback components.  **Retained—Incentives:** Reward/penalty components.  **Deleted:** Admin./workload items. |
| **Talent Management** | Rewarding (bonus, training, promotion); staff satisfaction (salary, career development); attracting talent | **Incentives**  (partially extracted) | Perceived fairness and strength of pay–performance and promotion linkages are key retention issues in Chinese primary care. Items directly related to rewards and career development satisfaction were extracted as incentive mechanisms. | **Retained:** Reward items (bonus, training, promotion, spiritual incentives) and satisfaction with salary/career development.  **Deleted:** Recruitment and administrative items. |

**3. Appendix C**

**Table S7. The dimensions and measurement items of goal setting:**

| Dimensions | Items |
| --- | --- |
| Goal clarity | GL1: the organizations goals are clear and unambiguous. |
|  | GL2: The organization has set long-term and short-term goals. |
|  | GL3: the organizations goals are easily to be understood |
|  | GL4: The future direction of the organization is clearly communicated to our team members. |
|  | GL5: Our team members have a good understanding of what our organization is trying to do. |
|  | GL6: If I have more than one goal to accomplish, I know which ones are most important and which are least important. |
|  | GL7: I have specific, clear goals to aim for on my job. |
| Goal attainment | GA1: The extent to which your working goals have been achieved in achieving your personal work goals  □0-20% □20%-40% □40%-60% □60%-80% □80%-100% |
|  | GA2: The likelihood of achieving your working goals being achieved  □0-20% □20%-40% □40%-60% □60%-80% □80%-100% |
|  | GA3: You have the competencies needed to achieve your working goals |
|  | GA4: You turst you can achieve working goals |
|  | GA5: It is easy for you to achieve these goals |
| goal identity | GI1: The goals in the organization are challenging but reasonable (neither too hard nor too easy). |
|  | GI2: Organizational members all agree on organizational goals |
|  | GI3: You fully agree with the organization's goals (9) |
|  | GI4: You fully agree with your personal work goals (13) |
|  | GI5: Clear organizational mission and goals give meaning and direction to work (7) |
| goal commitment | GC1: It’s hard to take this goal seriously. (R) |
|  | GC2: Quite frankly, I don’t care if I achieve this goal or not. (R) |
|  | GC3: I am strongly committed to pursuing this goal. |
|  | GC4: It wouldn’t take much to make me abandon this goal. (R) |
|  | GC5: I think this is a good goal to shoot for. |

**Table S8. The dimensions and measurement items of organizational centralization:**

| Dimensions | Items |
| --- | --- |
| Organizational centralization | OCE1: Before taking any action, you must ask your superiors |
|  | OCE2: When an emergency situation arises, action must be taken with the consent of superiors |
|  | OCE3: No action can be taken before obtaining approval from superiors |

**Table S9. The dimensions and measurement items of organizational culture:**

| Dimension | Items |
| --- | --- |
| organizational coordination | COO1: Members of different departments look at problems in different ways (R). |
|  | COO2: Members work at the same pace and have a tacit understanding. |
|  | COO3: It is easy to coordinate among departments. |
| organizational learning | OL1: Organizations view failure as an opportunity to learn and grow. |
|  | OL2: The organization encourages innovation and risk-taking and rewards them with certain rewards. |
|  | OL3: The organization often arranges learning activities. |
| organizational communication | OCO1: Generally, the purpose of organizational communication is clear  OCO2: Communication within the organization is smooth and effective  OCO3: Mistakes or wasted resources are often caused by poor communication in the workplace (R) |
| organizational change | OC1: The organization is changing, but there are no significant changes in the core work of the departments (R)  OC2: Change is difficult and often slow to move forward (R)  OC3: Different parts of the organization often work together to make changes |
| organizational feedback | OF1: The way our team does our job is rarely assessed (R).  OF2: Our team usually receives feedback on the quality of work we have done.  OF3: Our team members don’t have any idea how well we are doing our job (R). |
| procedural fairness | PF1: Equity in career development opportunities for members of the organization  PF2: Fairness in the scheduling of work for members of the organization  PF3: Fairness in the organization's performance evaluation process |
| competency development | CD1: The organization values the development of members' abilities  CD2: The organization conducts a series of trainings to enhance members' job skills  CD3: The organization member’s competence is improving |

**Table S10. The dimensions and measurement items of incentives:**

| Dimensions | Items |
| --- | --- |
| revenue incentive | RI1: The amount of income affects my motivation to work  RI2: I am willing to work hard for more income  RI3: The income gap with my colleagues will affect my work enthusiasm |
| assessment incentive | AS1: I accept the assessment indicators  AS2: I care about the results of my own appraisal  AS3: Appraisals make me feel pressure  AS4: I am willing to work hard to achieve various appraisal targets.  AS5: Appraisals are in my personal interest. |
| advancement incentive | AD1: I think advancement is very important  AD2: It is closely related to my personal interests.  AD3: It means better personal development  AD4: It can stimulate my enthusiasm for work  AD5: I can be promoted if I work hard. |

**Table S11. The dimensions and measurement items of quiet quitting:**

| Dimensions | Items |
| --- | --- |
| Detachment | DE1: I do the basic or minimum amount of work without going above and beyond.  DE2: If a colleague can do some of my work, then I let him/her do it.  DE3: I take as many breaks as I can.  DE4: I often pretend to be working in order to avoid another task. |
| Lack of initiative | LI1: I don’t express opinions and ideas about my work because I am afraid that the manager assigns me more tasks.  LI2: I don’t express opinions and ideas about my work because I think that working conditions are not going to change.  LI3: I often take initiative at your work. (R) |
| Lack of motivation | LM1: I find motives in my job. (R)  LM2: I feel inspired when I work. (R) |
